# Optic Neuropathy and Macular Ischemia Associated with Neurosarcoidosis: A Case Report

**DOI:** 10.4274/tjo.49799

**Published:** 2018-09-04

**Authors:** Burak Tanyıldız, Gizem Doğan, Nilüfer Zorlutuna Kaymak, Mehmet Engin Tezcan, Ahmet Kasım Kılıç, Sevda Şener Cömert, Aysu Karatay Arsan

**Affiliations:** 1 University of Health Sciences, Kartal Dr. Lütfi Kırdar Training and Research Hospital, Ophthalmology Clinic, İstanbul, Turkey; 2 University of Health Sciences, Kartal Dr. Lütfi Kırdar Training and Research Hospital, Rheumatology Clinic, İstanbul, Turkey; 3 University of Health Sciences, Kartal Dr. Lütfi Kırdar Training and Research Hospital, Neuorology Clinic, İstanbul, Turkey; 4 University of Health Sciences, Kartal Dr. Lütfi Kırdar Training and Research Hospital, Pulmonary Diseases Clinic, İstanbul, Turkey

**Keywords:** Macular ischemia, methotrexate, neurosarcoidosis, optic neuropathy

## Abstract

In this study, we present a case of bilateral optic neuropathy and macular ischemia in the right eye associated with neurosarcoidosis. A 26-year-old woman presented to our clinic with complaints of bilateral blurred vision. Bilateral granulomatous anterior uveitis, vitritis, optic neuropathy, and macular ischemia were detected in the right eye in slit-lamp examination. She also reported complaints of fever, weakness, sweating, arthralgia, and headache for 2 months. She was referred to the pulmonary diseases unit of our hospital due to hilar lymphadenopathy seen in her chest x-ray, and biopsies were taken for diagnostic purposes. Histological analysis of the mediastinal lymph node biopsies revealed chronic, non-caseating, granulomatous inflammation. Furthermore, the patient was referred to a neurologist due to concomitant complaint of intense headaches. She was diagnosed with neurosarcoidosis supported by findings on cranial magnetic resonance imaging and lumbar puncture. She received a 3-day course of high-dose (1 g/day) intravenous steroid treatment (methylprednisolone) followed by a tapering dose of oral prednisone. The patient began receiving oral methotrexate 15 mg/week as a steroid-sparing agent. Significant improvement in neurological and ophthalmological symptoms occurred in the first week of treatment. In this case report, we emphasized that neurosarcoidosis should be included in the differential diagnosis of patients with both bilateral optic neuropathy and macular ischemia. Furthermore, early diagnosis and timely treatment of neurosarcoidosis are important for favorable visual outcomes.

## Introduction

Sarcoidosis can affect multiple organs and is histologically characterized by non-caseified granulomas.^1^ Ocular involvement is observed in 25-50% of patients with systemic sarcoidosis.^2^ The most common findings are uveitis and conjunctival nodules.^3,4^

Neurosarcoidosis is found in 5-15% of those with systemic disease.^5,6^ In patients with neurosarcoidosis, ophthalmic symptoms are mostly associated with cranial nerve involvement and uveitis. The facial, trigeminal, oculomotor, and optic nerves are the most commonly involved cranial nerves.^7^

Presented herein is a patient with bilateral uveitis, unilateral macular ischemia, bilateral optic disc involvement, and a biopsy-confirmed diagnosis of pulmonary sarcoidosis. This case report emphasizes that optic disc involvement may be a sign of neurosarcoidosis in patients with sarcoidosis and that macular ischemia may develop due to reduced flow in the retinal artery resulting from inflammation.

## Case Report

A 26-year-old female patient presented to our clinic with blurred vision in both eyes for 2 weeks. She reported no history of any major illness, but had complaints of fever, sweating, exhaustion, joint pain, and headache starting 2 months earlier. Upon presentation to another center for these complaints, chest x-ray revealed hilar fullness and a thoracic computed tomography (CT) scan was performed. Thoracic CT revealed bilateral hilar lymphadenopathy, and the patient was referred to our hospital for further diagnosis and treatment, where she underwent endobronchial ultrasound-assisted lymph node biopsy in our pulmonary diseases unit. Histopathological examination revealed granulomatous structures, lymphocytes, and sporadic bronchial epithelial cells.

The patient was not under treatment when she presented to our clinic. Physically, she exhibited somnolence and clouding of consciousness. Her visual acuity was 20/25 in the right eye and 20/20 vision in the left eye. Granulomatous keratic precipitates and +1 Tyndall effect were detected in both eyes in anterior segment examination. In both eyes, +1 vitritis was observed in the anterior vitreous.

**Figure 1:**
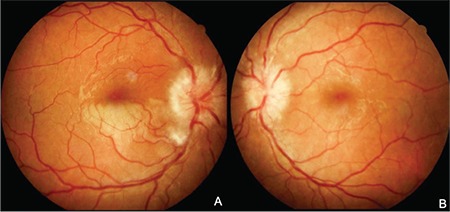
Color fundus photograph of a 26-year-old female patient at initial presentation: (A) Optic disc edema, soft exudate in the inferotemporal part of the optic disc, and ischemia in the inferior half of the macula is seen in the right eye; (B) optic disc edema is seen in the left eye

On fundus examination, the optic discs of both eyes were edematous and hyperemic. In the right eye, a soft exudate was observed inferior to the optic disc and the lower half of the macula was ischemic (Figures 1A, 1B). The patient was started on topical treatment with 1% prednisolone acetate 4 times daily and 0.5% tropicamide 3 times daily.

Neurology consultation was requested due to the patient’s complaints of intense headaches and the presence of bilateral optic disc involvement. However, no pathology was detected on neuromuscular examination. Cranial imaging and a lumbar puncture were performed. Diffusion magnetic resonance imaging (MRI) revealed extending nodular enhancements within and adjacent to the optic chiasm, at the basal cisternal level, and in the leptomeningeal layers in the posterior fossa. Thoracic and cervical MRI revealed leptomeningeal enhancement adjacent to the entire spinal cord. Cranial MR venography was normal.

**Figure 2:**
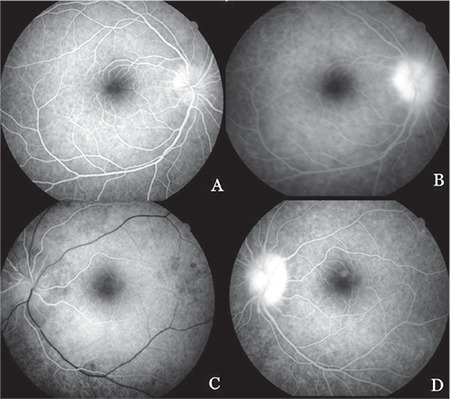
Fundus fluorescein angiography image obtained 2 weeks after initial presentation: (A-D) Optic disc hyperfluorescence beginning in the early phase and increasing in the late phase is seen in both eyes; (D) a hyperfluorescent spot increasing in intensity in the late phase is seen in the superior aspect of the left fovea

**Figure 3:**
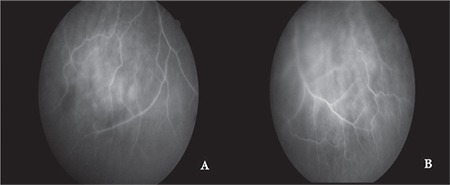
Peripheral fundus fluorescein angiography image shows leakage increasing in the late phase in the retinal vessels in the (A) right and (B) left eyes

Laboratory tests revealed low hemoglobin (10.7 g/dL; normal: 12-17), high sedimentation rate (57 mm/h; normal: 6-12), elevated CRP (9.4 mg/dL; normal: 0-3.5), and high urinary calcium (324 mg/24 hours; normal: 0-250). Anticardiolipin antibodies and lupus anticoagulants were negative. Cerebrospinal fluid (CSF) analysis showed high protein (145 mg/dL; normal: 15-45) and normal glucose (47 mg/dL; normal: 40-70), cell count in the cytological specimen was high (8500 cells/mL; normal: 0-10), and CSF pressure was normal (12 cm H_2_O; normal: 8-18). CSF tuberculosis culture and acid-fast bacilli staining was negative. Based on the laboratory values, lymph node biopsy, and imaging findings, the patient was diagnosed with neurosarcoidosis-associated optic neuropathy and treatment was initiated with intravenous 1 g methylprednisolone for 3 days followed by oral methylprednisolone at 1 mg/kg/day. The macular ischemia initially observed in the right eye regressed within 1 week. The patient’s general condition improved significantly with treatment and fundus fluorescein angiography (FFA) was performed. In both eyes, hyperfluorescence beginning in the early phase and increasing in the late phase was observed in the optic discs (Figures 2A-D) and a hyperfluorescent spot that increased in intensity in the late phase was observed on the vessel passing superiorly over the left fovea (Figure 2D). Hyperfluorescent leakage that increased toward the late phase was observed in the lower peripheral retinal vessels of both eyes (Figures 3A, 3B).

**Figure 4:**
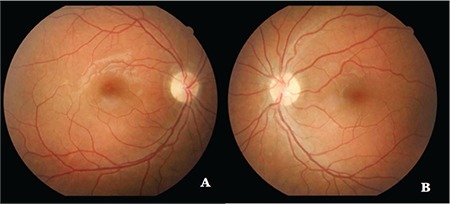
Fundus photograph taken 3 months after treatment: (A) Complete resolution of the optic disc edema and ischemia in the inferior macula is seen in the right eye; (B) Regression of the optic disc edema is seen in the left eye

Although systemic steroid therapy was reduced, it was decided to initiate systemic immunosuppressive therapy; oral methotrexate 15 mg/week and folic acid 5 mg/day were added to her treatment. Her vision was 20/20 in both eyes after 3 months of treatment. Anterior segment examination was normal. Fundus examination showed regression of the optic disc edema (Figures 4A, 4B). Follow-up diffusion MRI revealed that the extending nodular enhancements within and adjacent to the optic chiasm at the basal cisternal level and in the leptomeningeal layers in the posterior fossa had disappeared. In addition, thoracic and cervical MRI showed that the leptomeningeal enhancements along the spinal cord had completely resolved and the hilar lymphadenopathy had disappeared.

The patient had no systemic or ophthalmological recurrence while under oral 15 mg/week methotrexate and 5 mg/day folic acid therapy or during the 15-month follow-up period. No side effects related to systemic immunosuppressive therapy were observed.

## Discussion

Although the lungs are most commonly affected, sarcoidosis can manifest with extrapulmonary involvement including dermal, ocular, neurological, cardiac, renal, and gastrointestinal involvement.^8^ Neurological involvement is uncommon in sarcoidosis. Neurosarcoidosis is seen in 5-15% of patients with systemic sarcoidosis.^5,6^

Turner et al.^9^ reported that the central nervous system is also involved in 37% of patients with intraocular sarcoidosis. In a study by Menezo et al.^7^, it was found that 7.4% of patients with neurosarcoidosis had optic nerve involvement. In a series of 19 patients with systemic sarcoidosis, optic neuropathy was accompanied by granulomatous anterior uveitis in 10 patients, retinal vasculitis and cotton-wool spots in 2 patients each, and isolated vitritis, panuveitis, isolated choroidal involvement, macular exudates, and episcleritis in 1 patient each.^10^ In our case, the patient had systemic sarcoidosis with ocular involvement manifesting as bilateral anterior uveitis, vitritis, and macular ischemia in the right eye. When the patient underwent neurological evaluation for bilateral optic nerve involvement and headaches, we found that she also had neurosarcoidosis.

Yu and Yannuzzi^11^ associated bilateral decreased vision in a patient who had pulmonary neurosarcoidosis and neurosarcoidosis with findings of avascular zone enlargement on FFA and cystic changes in the macula on OCT. The patient was found to have bilateral perifoveal ischemia, which was presented as a rare finding of sarcoidosis involvement. Sarcoidosis usually causes non-ischemic retinal vasculitis.^12^ The macular ischemia in the right eye of our patient and the soft exudate in the lower temporal part of the optic disc may be attributable to reduced flow in the retinal artery due to inflammation. The macular ischemia and exudate regressed within the first week of treatment. Due to the poor general condition of the patient, OCT and FFA were not done during this period.

Unlike pulmonary sarcoidosis, spontaneous resolution of neurosarcoidosis is uncommon. Neurosarcoidosis-related morbidity and mortality are minimized with treatment.^13^ Although corticosteroids appear to be the first choice for the treatment of neurosarcoidosis, response rates to treatment with corticosteroids alone are lower in patients with neurosarcoidosis compared to patients with pulmonary sarcoidosis.^14,15^ In the long-term treatment of neurosarcoidosis, reactivation has been reported when the corticosteroid dose is reduced to 20-25 mg.^15^ The addition of immunosuppressive agents such as methotrexate,^14^ azathioprine,^16^ mycophenolate mofetil,^16^ and chlorambucil^17^ to corticosteroid therapy has been reported in case reports. There are also studies in which the anti-TNF inhibitor infliximab has been used as a biological agent.^18^ In one report, a 61% remission rate was achieved in neurosarcoidosis by discontinuing corticosteroids and treating with methotrexate.^14^ In the present case, full remission with no recurrence during the 15-month follow-up period was achieved with methotrexate treatment.

In conclusion, neurosarcoidosis should be included in the differential diagnosis of patients with bilateral optic neuropathy and uveitis. Although macular ischemia is a rare finding of sarcoidosis, we should bear in mind that it may arise as a result of reduced flow in the retinal artery due to inflammation. Early diagnosis and treatment improve the prognosis of the disease and of ocular involvement if present. Sarcoidosis requires a multidisciplinary approach, and ophthalmologists play a key role in diagnosis and treatment planning.
